# Ultrasound-guided versus blind arthrocentesis in knee osteoarthritis: A systematic review and meta-analysis

**DOI:** 10.1097/MD.0000000000041389

**Published:** 2025-01-31

**Authors:** Xiaoyan Deng, Yamei Li, Daishun Li

**Affiliations:** a Department of Rehabilitation Medicine, Chengdu Tianhui Community Health Service Center, Sichuan, China; b Department of Rehabilitation Medicine, Sichuan Provincial People’s Hospital, University of Electronic Science and Technology of China, Sichuan, China.

**Keywords:** arthrocentesis, knee osteoarthritis, systematic review, ultrasound

## Abstract

**Background:**

To summarize the current evidence about effectiveness and accuracy of using ultrasound-guided compared to blind arthrocentesis in the treatment of knee osteoarthritis.

**Methods:**

Web of Science, the Cochrane Central Register of Controlled Trials, EMBASE, Scopus, PubMed, ClinicalTrials.gov, Wangfang Database, and SinoMed were conducted from their inception to February 2024. Eligible studies included Randomized controlled trials (RCTs) and non-RCTs that compared the ultrasound-guided and blind arthrocentesis in knee osteoarthritis, with outcomes assessed base on pain, function, accuracy, and additional factors such as satisfaction, cost-effectiveness, fluid yield, and synovial membrane thickness.

**Results:**

Twenty-one studies that met the inclusion criteria (1924 patients) were identified. The results indicated that ultrasound-guided arthrocentesis was superior to blind arthrocentesis (10 trials; MD = −0.37; 95% CI = −0.55 to −0.19; *P* = .000). However, no significant difference was found in function improvement (7 trials; SMD = −0.60; 95% CI = −1.31 to 0.12; *P* = .101). Ultrasound-guided arthrocentesis also demonstrated better accuracy compared to blind arthrocentesis (RR = 1.26, 95% CI: 1.09–1.46, *P* = .001). For satisfaction, the result reported ultrasound was better than the blind group (MD = 1.11; 95% CI = 0.67–1.54; *P* = .000) at immediate post-procedure, and at the 4 to 6 weeks (MD = 0.98; 95% CI = 0.56–1.41; *P* = .000).

**Conclusion:**

In the comprehensive systematic review and meta-analysis of knee osteoarthritis, ultrasound-guided arthrocentesis is superior to anatomic landmark–guided arthrocentesis in terms of pain reduction and accuracy.

## 1. Introduction

Knee osteoarthritis is a widespread degenerative musculoskeletal disease. It encompasses conditions such as knee osteoarthritis, rheumatoid arthritis, traumatic arthritis, gouty arthritis and others.^[1]^ It usually occurs in the elderly, obese people, and people with lower limb injuries.^[2]^ Knee osteoarthritis involves both inflammation and structural changes in the joints.^[3]^ It is a predominant cause of severe knee pain, disability, and leading to a decline in quality of life.^[4–6]^ In clinical practice, knee arthrocentesis is routinely performed for this condition, typically by orthopedic surgeons, rheumatologists, and physiotherapists.^[6,7]^ Consequently, it places a huge burden on healthcare systems globally.^[8]^ For the management of knee pain, treatments such as hyaluronic acid (HA) injections, corticosteroid injections, or physical exercise are recommended.^[9,10]^ Intra-articular injection or aspiration has been used in the treatment of knee pain for a long time and has demonstrated its effectiveness.^[11–14]^ Arthrocentesis using common anatomical landmarks is a frequently employed method. Nevertheless, graphically guided (radiographic or ultrasound) arthrocentesis has been demonstrated to offer advantages for the knee, including pain reduction and improved function.^[15,16]^

With the development of ultrasound imaging technology, the clinical application of ultrasound-guided injections in treating knee osteoarthritis is becoming increasingly widespread, and its efficacy is continuously improving.^[17,18]^ Under ultrasound guidance, healthcare providers can observe the articular fluid, articular surface, and other anatomical structures, while simultaneously monitoring the position of the puncture needle dynamically. Hence, it offers enhanced accuracy and controllability. Ultrasound-guided injection can greatly enhance the accuracy and effectiveness of knee cavity injections, without increasing or even reducing the pain of associated with the procedure.^[19–22]^

While several systematic reviews have demonstrated improved knee accuracy, and some systematic reviews describe the effectiveness of ultrasound-guided and blind injection in other joints such as shoulder.^[23–26]^ However, previous literature has not conducted a comprehensive evaluation comparing ultrasound-guided and blind knee arthrocentesis. Therefore, we conducted a systematic review and meta-analysis to comprehensively assess the clinical efficacy of ultrasound-guided vs blind knee arthrocentesis, encompassing outcomes such as efficacy, accuracy, satisfaction, cost-effectiveness, fluid obtained, and thickened synovial membrane.

## 2. Methods

### 2.1. Protocol and registration

This study adhered to the guidelines outlined by the Cochrane Collaboration and was reported in accordance with the Preferred Reporting Items for Systematic Reviews and Meta-analyses guidelines.^[27]^ The protocol for this study was registered on PROSPERO’s web platform (CRD42022308396).

### 2.2. Search strategy

Web of Science, the Cochrane Central Register of Controlled Trials, EMBASE, Scopus, PubMed, ClinicalTrials.gov, Wangfang Database, and SinoMed were conducted from their inception to February 2024 using search terms knee osteoarthritis (OA)/knee OA and injection. We presented the retrieval method in Table S1, Supplemental Digital Content, http://links.lww.com/MD/O308. We also filtered the references of included reviews and trials to recognize potentially feasible researches. Open Grey (http://www.opengrey.eu/) was explored.

### 2.3. Inclusion and exclusion criteria

#### 2.3.1. Types of studies

Randomized controlled trials (RCTs) or non-RCTs that met the inclusion criteria compared to evaluate ultrasound-guided vs blind arthrocentesis for treating knee osteoarthritis. If the study is incomplete, we will attempt to contact the corresponding author by email to obtain the full text. However, if we are unable to receive a response, the study will be excluded from consideration.

#### 2.3.2. Types of participants

Participants who underwent knee injections or aspiration were adults (18 years of age or older) diagnosed with knee osteoarthritis, without restrictions based on gender or nationality. The exclusion criteria were patients who were not diagnosed with knee osteoarthritis, such as those with knee inflammation or pain following trauma or surgery.

#### 2.3.3. Types of Interventions

The intervention was ultrasound-guided arthrocentesis, while the comparator was blind arthrocentesis. We did not limit patients based on specific clinical diagnostic criteria or the type of injected drugs.

#### 2.3.4. Types of outcome measures

The primary outcome was the assessment of pain using visual analogue scale (VAS). The secondary outcomes comprised functionality, primarily assessed using the Western Ontario and McMaster Universities Osteoarthritis Index (WOMAC), precision, satisfaction, cost-effectiveness, volume of obtained fluid, and thickened of the synovial membrane. The outcomes were ranked according to recommendations by Juhl et al.^[28]^ Pain or functional outcomes were measured by different scales (Table S2, Supplemental Digital Content, http://links.lww.com/MD/O308).

### 2.4. Study selection

In this review, search results were imported into EndNote for screening.

A 3-stage screening approach was employed to sift through relevant studies First, all titles were screened for inclusion criteria by 1 reviewer, and articles that did not meet the requirements were excluded. Second, another reviewer examined the titles and abstracts of each researches, independently. Third, 2 authors reviewed the full range of articles to assess the inclusion criteria for each study that might meet the inclusion criteria. Any disagreements that arose were resolved through discussion.

### 2.5. Data collection

Two reviewers obtained the necessary data from included researches with a normalized chart, involving the lead author, year of issued, country, participants, total number of samples, intervening method, follow-up, and results, independently. The extracted data were cross-checked by 2 authors. For missing data, we send emails to the corresponding author or estimated the mean, confidence interval (CI).^[29]^ All inconsistencies were resolved by consulting.

### 2.6. Risk of bias

We used the Cochrane Collaboration’s 2.0 tool (RoB 2)^[30]^ for randomized trials to appraise the risk of bias in the following domains: randomization process, deviations from intended interventions, missing outcome data, measurement of the outcome, selection of the reported results, and overall bias. The risk of bias in each domain was judged as “low,” “some concerns,” or “high.” The Risk Of Bias in Non-randomized Studies of Interventions (RoB-I)^[31]^ was employed for non-RCTs. The RoB-I comprised the following domains: confounding, participant selection, intervention classification, deviations from intended interventions, missing data, outcome measurement, and selection of reported results. The Grades of Recommendation Assessment, Development, and Evaluation (GRADE) tool was used to evaluate the quality of the evidence.^[32]^ Disagreements were resolved by reference to the original text and/or by discussion.

### 2.7. Data analysis

All data analyses were calculated by Revman 5.4 and STATA 15.0. The combined effect was evaluated according to the final results. The scores in pain, and function were expressed as mean differences with 95% CIs. If the evaluation scales were different, the standard MD (SMD) was calculated. The accuracy outcome (number of accuracy), and the risk ratios with 95% CIs were used. We tested for heterogeneity in the pooled results using *I*^2^ values as follows: *I*^2^ > 50% was defined as significant heterogeneity; *I*^2^ < 50% was defined as no significant heterogeneity. A fixed-effects model was used to combine studies with *I*^2^ < 50%. Otherwise, random effects model were used. For publication bias, Funnel plot is used. We use Begg or Egger test evaluate the asymmetry of the funnel plot. Trim and fill analysis was also conducted.

### 2.8. Trial sequential analysis

We performed TSA to explore whether our evidence meta-analyses are reliable and conclusive.^[33,34]^ With the TSA software (version 0.9.5.10) to maintain the type I error (α) and type II error (β) in cumulative meta-analyses, with an α of 5% and a β of 20% (power of 80%) based on O’Brien–Fleming spending functions.

### 2.9. Sensitivity and subgroup analyses

We conducted sensitivity analyses to assess the robustness of the analysis results. Furthermore, subgroup analysis was conducted, stratified by efficacy at different follow-up durations (immediately-1 month, 1–3 months, 3–6 months), and different types of arthrocentesis of drugs (corticosteroids, HA).

## 3. Results

### 3.1. Study characteristics

**Figure 1.**
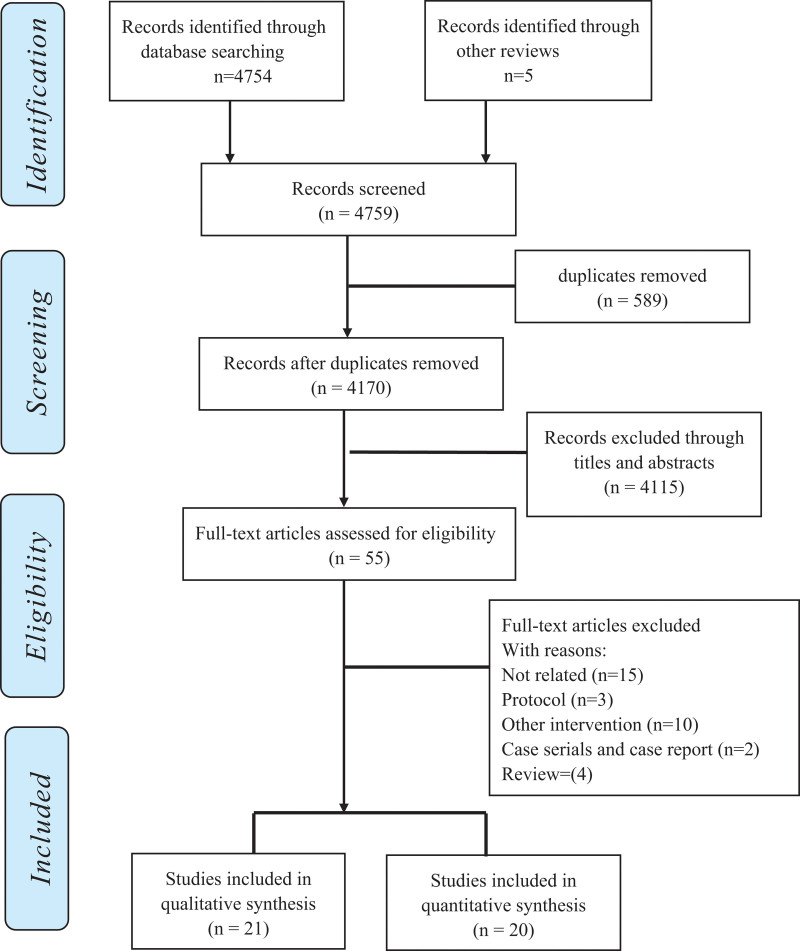
Flow diagram of study selection.

In total, 4754 studies were achieved. Of these, 4754 studies were screened in the initial databases. From the issued systematic reviews, 5 additional studies were selected. After removing the duplicate studies and reviewing the abstracts and titles, 55 studies were ultimately included. Finally, 21 studies met the inclusion criteria and were included in the data analysis (Fig. 1). In total, 1924 participants were included in the study (ultrasound-guided, n = 977; blind, n = 947). The sample size for each study varied, ranging from 12 to 156 participants per group, with an average age of 18 to 80 years. Follow-up for enrollment ranged from immediately to 6 months. In Table 1, we presented the characteristics of the studies that met the requirements. Twelve researches were related to pain, of which ten studies^[17,18,20–22,35–41]^ based on visual analog scale (VAS) and 2 studies^[42,43]^ reported the pain based on ratio. Functional outcomes were evaluated in 7 trials^[17,18,20,22,38,40,44]^ and 2 studies^[42,43]^ reported the efficiency of function. Accuracy was reported in ten study.^[21,35,39,45–50]^ Three studies^[35,37,43]^ reported a comparison of the fluid obtained. Two studies^[20,40]^ reported satisfaction of patients. One study^[43]^ reported the thickened synovial membrane. One study^[36]^ has shown the costs of ultrasonography for hospital outpatient guidance.

**Table 1:** The characteristics of studies.

Author year country	No. of patients	Women, no. (%)	Mean age (SD) /media (min–max)	Type of intervention and dose	Follow-up	Outcomes measure
Sang 2009 Korea	45	34/75	60.6 ± 7.9	Sonographically-guided injection: 2 mL of hyaluronic acid	After injection	Accuracy
	44	31/70	59.6 ± 9.9	Blind injection:2 mL of hyaluronic acid		
Jennifer L 2010 USA	39	15 (38)	54.5	Sonographically-guided aspiration: joint fluid	Immediately	Accuracy VAS Technique was easier to perform procedure time Fluid obtained
	27	8 (30)	50.5	Blind aspiration: Joint fluid		
Smith 2010 USA	12	None	None	Ultrasound-guided Injection:1.5 mL of 50% water-diluted colored latex	After injection	Accuracy
	12	None	None	Blind injection:1.5 mL of 50% water-diluted colored latex		
Sibbitt W 2011 USA	46	None	None	Sonographic injection: 80 mg tri-amcinolone acetonide	2 wk 6 mo	VAS cost-effectiveness
	46	None	None	Blind injection: 80 mg tri-amcinolone acetonide		
Curtiss H 2011 USA	20	14/6	75.7 ± 14.5	Sonographic guided injection: 50% diluted	After injection	Accuracy
	20	14/6	75.7 ± 14.5	Blind injection:50% diluted		
Bum 2012 Korea	50	36/72	60.2 ± 8.1	Ultrasound-guided injection: hyaluronic acid 2 mL	After injection	Accuracy
	49	35/71.4	59.8 ± 7.9	Blind injection: hyaluronic acid 2 mL		
Sibbitt W 2012 Australia	42	None	None	ultrasound (US)-guided injection: corticosteroid	2 wk	VAS Aspirated synovial fluid
	22	None	None	Blind injection: corticosteroid		
Jang,S 2013 Korea	44	35/44	61.48	Ultrasound-guided in-plain (IP): 1% lidocaine (1 mL), 20 mg of tri-amcinolone (1 mL), and a nonionic contrast agent (4 mL)	After injection	Accuracy
	41	31/41	62.02	Ultrasound-guided in-plain (OOP):1% lidocaine (1 mL), 20 mg of tri-amcinolone (1 mL), and a nonionic contrast agent 4 mL).		
	41	33/41	61.10	Blind:1% lidocaine (1 mL), 20 mg of tri-amcinolone (1 mL), and a nonionic contrast agent 4 mL)		
Hosseini 2016 Iran	100	73/73	64.46 ± 7.16	Ultrasound-guided injection: 4 mL hyaluronic acid	Immediately	Accuracy
	123	90/73	63.68 ± 7.54	Blind injection: 4 mL hyaluronic acid		
Qing Y 2017 China	50	None	61 ± 20.5	Ultrasound-guided injection: 2 mL hyaluronic acid	5 wk	Pain Function Fluid obtained Synovial thickness
	50			Blind injection: 2 mL hyaluronic acid		
Kianmehr 2018 Iran	31	48/78.6	61.52 ± 9.0	Ultrasound-guided injection: 2 mL hyaluronic acid	6 wk 12 wk	VAS WOMAC KOOS
	30			Blind injection: 2 mL hyaluronic acid		
☐	22	20/22	63.40 ± 6.20	Blind Injection: 20 mg of tri-amcinolone acetonide and 1 mL of 1% lidocaine		
Quanbing 2018 China	46	24/46	59.42 ± 2.36	• Ultrasound (US)-guided injection: betamethasone 1 mL + normal saline 2 mL + lidocaine 1 mL	1 wk 4 wk	Pain Function
	46	23/46	59.17 ± 2.28	Blind injection: betamethasone 1 mL + normal saline 2 mL + lidocaine 1 mL		
Yubing X 2018 China	34	None	18 to 45	ultrasound (US)-guided injection: platelet rich plasma	1 mo 6 mo	VAS Lysholm knee score
	34	None	18 to 45	Blind injection: platelet rich plasma		
Lee J 2019 Korea	25	23/25	65.36 ± 7.12	Sonographic guided Injection: 20 mg of tri-amcinolone acetonide and 1 mL of 1% lidocaine	1 wk 4 wk	VAS Accuracy
☐	22	20/22	63.40 ± 6.20	Blind Injection: 20 mg of tri-amcinolone acetonide and 1 mL of 1% lidocaine		
Sheth T 2020 USA	19	14 (73.7)	60.89 ± 10.4	Sonographic: 40 mg of depomedrol and 3 cc of 1% lidocaine	Immediately 4 to 6 weeks	VAS KOOS WOMAC Likert scale Satisfaction
	18	12 (66.7)	58.27 ± 9.64	Blind Injection: 40 mg of depomedrol and 3 cc of 1% lidocaine		
Cankurtaran 2020 Turkey	11	None	50 to 80	Sonographic guided: 2% lidocaine and 20 mg of tri-amcinolone	1 mo 3 mo	VAS WOMAC NHP The timed up and go test 6-min walk test 30-s chair stand test SCT
	20	None	50 to 80	Blind injection: 2% lidocaine and 20 mg of tri-amcinolone		
Yanxia F 2020 China	51	22/51	52.15 ± 9.30	• ultrasound (US)-guided: not mentioned	1 mo	Accuracy
	51	19/51	53.01 ± 9.35	• Blind injection: not mentioned	3 mo	Efficacy
Ferran 2020 Spain	156	None	None	Ultrasound -guided injection: 1 cc of colored natural latex		Accuracy
	156	None	None	ultrasound -guided injection: 1 cc of colored natural latex		
Dongmei J 2021 China	30	22/30	59.7 ± 10.1	ultrasound-guided injection: 1 mL of methadone and 3 mL of 1% lidocaine Blind injection:1 mL of methadone and 3 mL of 1% lidocaine	Immediately 5wk	VAS KOOS Satisfaction
	29	19/29	61.2 ± 9.2	Blind injection: 1 mL of methadone and 3 mL of 1% lidocaine		
Songyang Z 2021 China	60	38/60	64.46 ± 2.33	• ultrasound -guided injection: Tri-amcinolone acetonide ≤ 40 mg + water for injection + 2% lidocaine	6 mo	Lequesne rating scale KDC knee score table
	60	40/60	64.55 ± 2.41	Blind injection: sodium hyaluronate 2 mL		
Li Z 2021 China	25	20/80	60.16 ± 4.13	Ultrasound-guided injection: Hyaluronic acid 2 mL for 4 times	1 mo 3 mo	VAS WOMAC
	25	21/84	58.44 ± 4.44	Blind injection: hyaluronic acid 2 mL for 4 times		

Abbreviations: KOOS = knee injury and osteoarthritis outcome score, NHP = the Nottingham health profile, SCT = stair climb test, VAS = visual analogue scale, WOMAC = Western Ontario and McMaster Universities osteoarthritis index.

- **Quality assessment**

The assessment of bias risk of included studies is provided in the Figures S1 and S2, Supplemental Digital Content, http://links.lww.com/MD/O309. Eighteen RCTs were assessed using RoB 2; and the domain with the lowest risk of bias was “randomization process,” and that with some concerns risk of bias was “selection of the reported result”. Regarding the overall risk of bias, 8 studies were ranked low, 9 as some concern, and 1 as high (Figure S1, Supplemental Digital Content, http://links.lww.com/MD/O309). Additionally, 3 non-RCTs were assessed using RoB-I; the domain with the lowest risk of bias was “measurement of outcome”; that with the highest risk was “selection of participants”. As for the overall risk of bias, 2 studies were ranked moderate, and 1 was critical (Figure S2, Supplemental Digital Content, http://links.lww.com/MD/O309). According to the GRADE system, the evidence quality of the included studies was low, or moderate. The evidence was downgraded due to various factors, including the risk of bias, imprecision, and inconsistency. We put the GRADE results in Table 2.

**Table 2:** Results of GRADE.

Certainty assessment							No. of patients		Effect		Certainty
No. of studies	Study design	Risk of bias	Inconsistency	Indirectness	Imprecision	Publication bias	US	BLIND	Relative (95% CI)	Absolute (95% CI)	
Pain (assessed with VAS; scale from 0 to 10)											
12	Randomized trials	Serious*	Not serious	Not serious	Not serious	None	395	337	-	SMD 0.37 SD lower (0.55 lower to 0.19 lower)	⨁⨁⨁◯ Moderate
Function (scale from 0 to 100)											
9	Randomized trials	Serious*	Serious†	Not serious	Not serious	None	307	303	-	SMD 0.38 SD fewer (1.23 fewer to 0.47 more)	⨁⨁◯◯ Low
Accurately (follow-up: range 1 d to 6 mo; assessed with: RR)											
10	Randomized trials	Serious*	Not serious	Not serious	Not serious	None	499/565 (88.3%)	324/525 (61.7%)	RR 1.15 (1.03–1.29)	93 more per 1000 (from 19 more to 179 more)	⨁⨁⨁◯ Moderate
Fluid obtained (assessed with: mL)											
3	Randomized trials	Not serious	Not serious	Not serious	Serious‡	None	115	83	-	MD 0.09 higher (0.19 lower to 0.38 higher)	⨁⨁⨁◯ Moderate
Satisfaction (assessed with: Likert scale; scale from 0 to 5)											
2	Randomized trials	Not serious	Not serious	Not serious	Serious‡	None	49	47	-	MD 0.98 higher (0.56 higher to 1.41 higher)	⨁⨁⨁◯ Moderate

Abbreviations: CI = confidence interval, MD = mean difference, RR = risk ratio, SMD = standardized mean difference.

* Downgraded 1 level for risk of bias.

† Downgraded 1 level for inconsistency: *I*^2^ = 93.7%.

‡ Potential imprecision due to availability of small size.

### 3.2. Effect of intervention: pain relief

**Figure 2.**
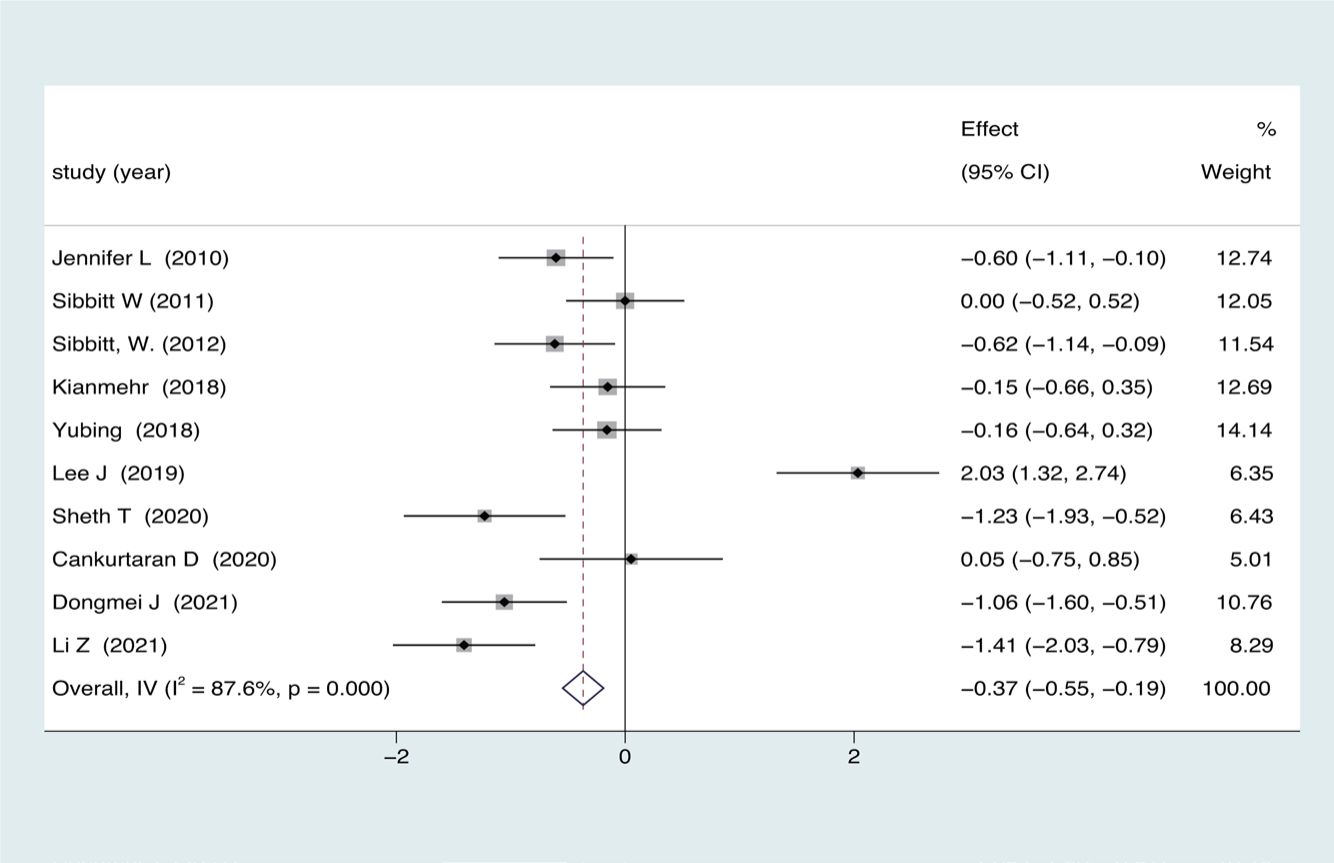
Forest plot for effects of ultrasound-guided vs blind arthrocentesis for pain.

Ten trials (540 participants) were included for quantitative analysis based on VAS. Moderate certainty evidence indicated a significant effect of ultrasound guidance on pain reduction (MD = −0.37; 95% CI = −0.55 to −0.19; *P* = .000), with significant heterogeneity (*I*^2^ = 87.6%, *P* = .000; Fig. 2) at the final follow-up. In the subgroup analysis based on follow-up periods, a statistically significant difference was observed between ultrasound-guided vs blind arthrocentesis for the duration of 1 to 3 months (MD = −0.97, 95% CI = −1.25 to −0.70, *P* = .000, Table 3). In the subgroup analysis based on the type of drugs used in arthrocentesis, the findings revealed that ultrasound-guided was superior to blind arthrocentesis when utilizing corticosteroids and HA, respectively (6 studies: MD = −0.30, 95% CI = −0.55 to −0.05, *P* = .007; two studies, MD = −0.65, 95% CI = −1.04 to −0.20, *P* = .000, Table 3).

**Table 3:** The subgroup of pain and function for ultrasound vs blind.

	Pain			Function		
	n	Effect size (95% CI)	*P*	n	Effect size (95% CI)	*P*
Follow-up periods						
Immediately-1 mo	7	−0.50 (−1.22, 0.22)	.174	2	−0.31 (−1.29, 0.67)	.534
1 to 3 mo	5	−0.97 (−1.25, −0.70)	.000	6	0.62 (−0.53, 1.76)	.29
3 to 6 mo	2	0.00 (−0.35, 0.35)		3	−0.93 (−2.18, 0.32)	.146
Arthrocentesis of drugs						
Corticosteroids	2	−0.65 (−1.04, −0.26)	.020	4	−0.51 (−1.78, 0.76)	.453
Hyaluronic acid	6	−0.30 (−0.55, −0.05)	.001	2	−0.98 (−1.41, −0.54)	.000

### 3.3. Effect of intervention: function improvement

**Figure 3.**
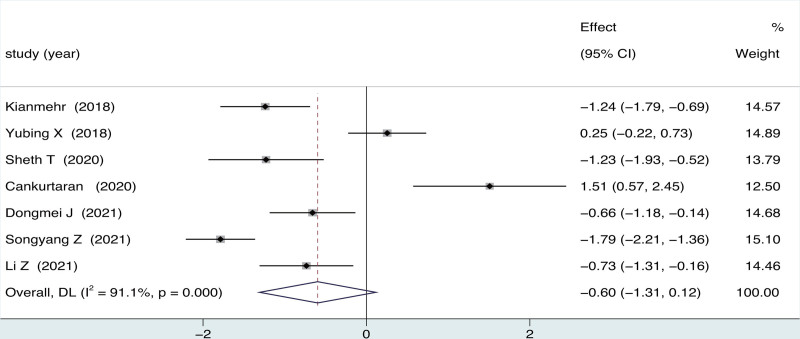
Forest plot for effects of ultrasound-guided vs blind arthrocentesis for function.

A total of 7 trials (418 patients) were contained in the analysis. Low certainty evidence suggested that there was no statistical significance difference in functional improvement between ultrasound-guided and blind arthrocentesis (SMD = −0.60, 95% CI = −1.31 to 0.12; *P* = .379), accompanied by substantial heterogeneity (*I*^2^ = 91.1%, *P* = .000; Fig. 3). In the subgroup analysis based on follow-up periods, no statistically difference was observed between ultrasound-guided and blind arthrocentesis across all follow-up durations (Table 3). In the subgroup analysis based on the arthrocentesis of drugs during arthrocentesis, a statistically significant difference was noted between ultrasound-guided and blind group for HA (two studies, SMD = 0.98; 95% CI = −1.41 to −0.54; *P* = .000), accompanied by significant heterogeneity(*I*^2^ = 17.6%, *P* = .271) (Table 3).

### 3.4. Accurately

**Figure 4.**
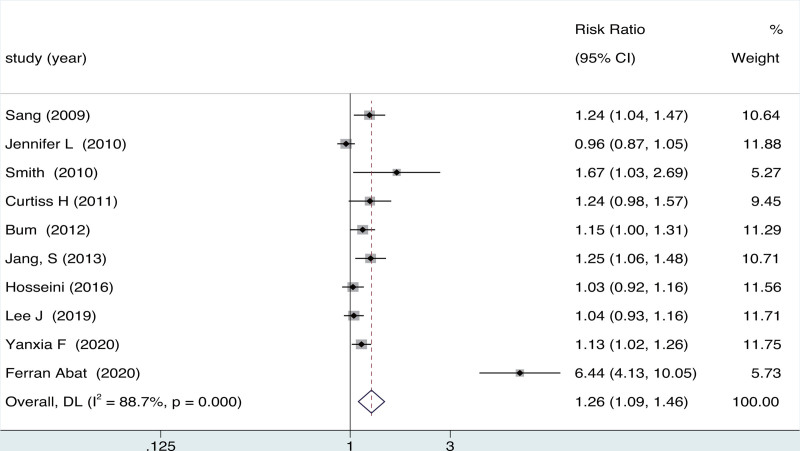
Forest plot for effects of ultrasound-guided vs blind arthrocentesis for accuracy.

Ten studies (1088 participants) provided the accuracy on ultrasound-guided vs blind group. A statistically significant difference was observed between the ultrasound-guided and blind group (RR = 1.26, 95% CI = 1.09 to 1.46; *P* = .001), accompanied by significant heterogeneity (*I*^2^ = 72.4%, *P* = .000) (Fig. 4).

### 3.5. Other outcomes

**Figure 5.**
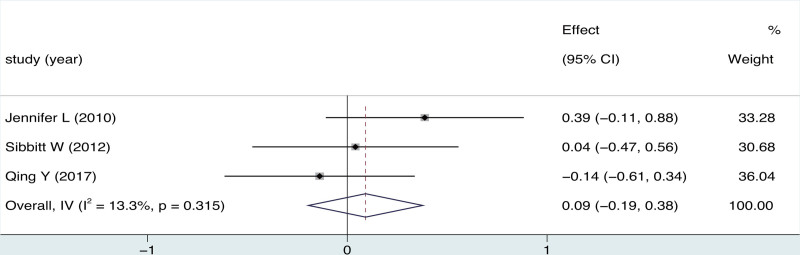
Forest plot for effects of ultrasound-guided vs blind arthrocentesis for fluid obtained.

**Figure 6.**
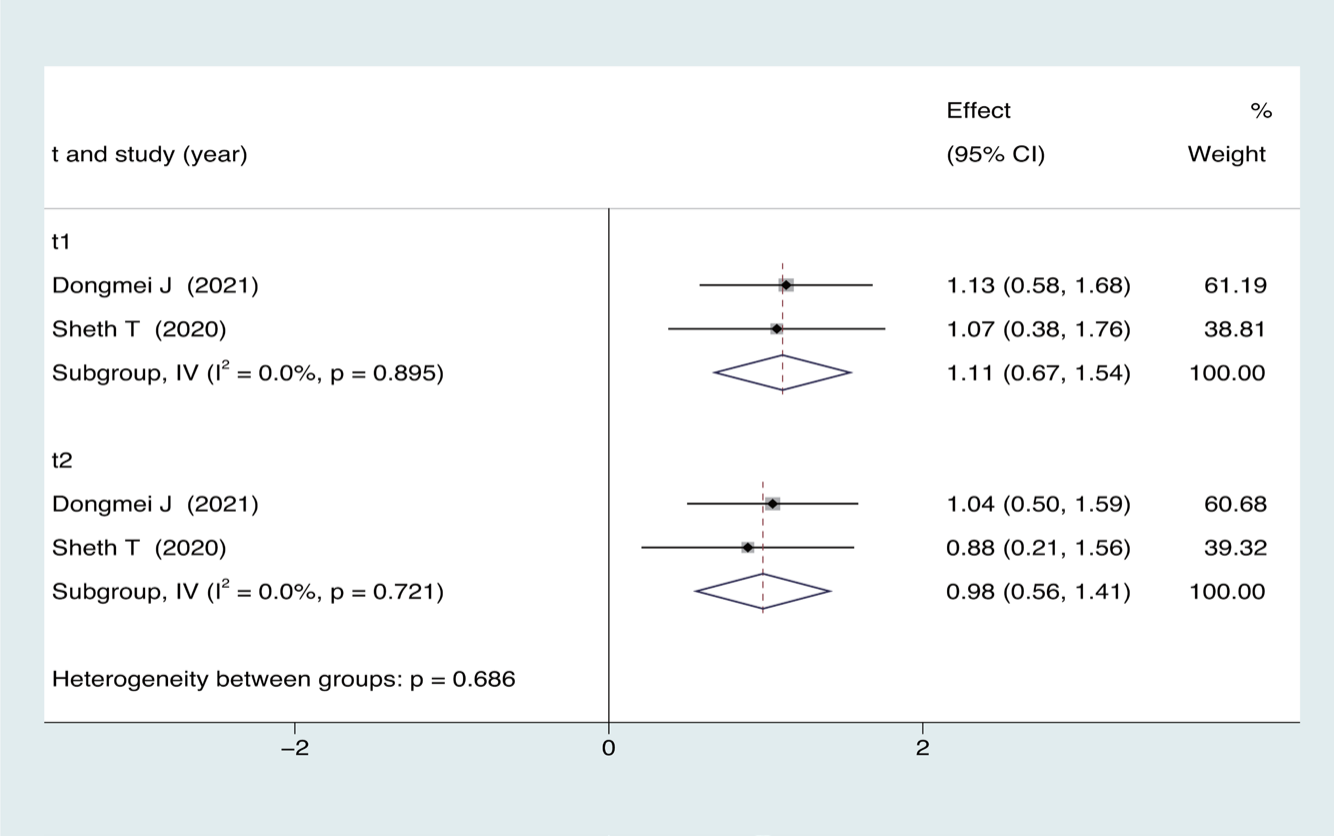
Forest plot for effects of ultrasound-guided vs blind arthrocentesis for satisfaction.

Only 1 study^[36]^ has demonstrated that the utilization of ultrasonography can slightly reduce costs by 13%($17) per patient per year compared to blind (*P* < .13). Additionally, it significantly decreases the cost per patient per year by 58% ($224) (*P* < .0001) for hospital outpatient clinics. There were 3 studies^[35,37,43]^ reported the mean volume of fluid obtained comparing ultrasound-guided and blind arthrocentesis. The pooled analysis revealed no significant complications in either group (3 trials; MD = 0.09; 95% CI = −0.19 to 0.38; *P* = .529), with significant heterogeneity (*I*^2^ = 13.3%, *P* = .315; Fig. 5). One study^[43]^ reported that ultrasound-guided was superior to blind arthrocentesis in reducing the thickened synovial membrane. In terms of patient satisfaction measured on the Likert scale, 2 study^[20,40]^ indicated that the ultrasound-guided group outperformed the blind group (MD = 1.11; 95% CI = 0.67 to 1.54; *P* = .000) both immediate post-procedure and at the 4 to 6 weeks (MD = 0.98; 95% CI = 0.56 to 1.41; *P* = .000) (Fig. 6).

### 3.6. Sensitivity analysis

We conducted sensitivity analyses to assess the impact of individual studies on the overall effect size by removing each variable and recalculating the pooled effect size. Only data from studies with more than 2 studies were pooled were included in pooled analysis. Following the exclusion of 1 study at a time, the overall outcome remained consistent, indicating stability in the results (Figures S3–S7, Supplemental Digital Content, http://links.lww.com/MD/O309).

### 3.7. Publication bias

We use Funnel plots to detect publication bias for all outcomes. Visual inspection of funnel plots did not reveal obvious evidence of asymmetry for pain and function (Figures S8 and S9, Supplemental Digital Content, http://links.lww.com/MD/O309). We use Egger test (*P* = .63), and Begg test (*P* = 1.00) for pain (Figures S10 and S11, Supplemental Digital Content, http://links.lww.com/MD/O309), and Egger test (*P* = .240), Begg test (*P* = .548) for function (Figures S12 and S13, Supplemental Digital Content, http://links.lww.com/MD/O309). Visible evidence of asymmetry appeared in the funnel plot for accuracy (Figure S14, Supplemental Digital Content, http://links.lww.com/MD/O309). We use the Egger test (*P* = .004), and Begg test (*P* = .007) (Figures S15 and S16, Supplemental Digital Content, http://links.lww.com/MD/O309). Trim and fill computation did not change the result for all results (Figures S17–S19, Supplemental Digital Content, http://links.lww.com/MD/O309).

- **Trial sequential analysis**

When the probability α = 0.05 (type I error) and probability β = 0.2 (the type II error) were set, TSA was performed according to the pain results. TSA analysis conducted on pain demonstrated that ultrasound-guided arthrocentesis yielded superior results compared to blind arthrocentesis (Figure S20, Supplemental Digital Content, http://links.lww.com/MD/O309). TSA analysis of function showed that there was no obvious difference in the effectiveness between the ultrasound-guided and blind arthrocentesis (Figure S21, Supplemental Digital Content, http://links.lww.com/MD/O309). The accuracy curve of treatment crossed the boundary between the traditional value and the TSA value, and the cumulative value of information quantity exceeded the expected information quantity line (TSA = 581). We can conclude that the accuracy of ultrasound-guided was superior to that of blind arthrocentesis (Figure S22, Supplemental Digital Content, http://links.lww.com/MD/O309).

## 4. Discussion

This systematic review examined the effects of ultrasound-guided vs blinded arthrocentesis on knee osteoarthritis, focusing on pain, function, accuracy, satisfaction, cost-effectiveness, volume of fluid obtained, and reduction in thickened synovial membrane. As of now, there hasn’t been comprehensive systematic review assessing the comparative effectiveness of ultrasound-guided vs blind arthrocentesis specifically for knee osteoarthritis. Our systematic review has identified 21 studies involving 1924 patients. The findings from the systematic review indicate that ultrasound-guided arthrocentesis, in comparison to blind arthrocentesis, proves to be more effective in alleviating pain and increasing accuracy in managing knee osteoarthritis. Subgroup analyses revealed that ultrasound-guided arthrocentesis exhibited superiority over blind arthrocentesis in alleviating pain within a duration of 1 to 3 months. Additionally, ultrasound-guided were found to be more effective in relieving pain and improving function compared to blinded arthrocentesis, particularly in cases involving HA. Simultaneously, ultrasound-guided were observed to provide greater pain relief compared to blinded arthrocentesis in corticosteroids. The results indicated that ultrasound-guided offered superior accuracy compared to blind arthrocentesis. Additionally, concerning patient satisfaction, the findings revealed that the ultrasound-guided exhibited high satisfaction levels than the blind arthrocentesis both immediate post-procedure and at the 4 to 6 weeks. Caution is warranted when interpreting the results due to the limited number of included studies and sample sizes. These results may necessitate consideration of the following factors: Firstly, ultrasound-guided knee cavity injections may offer improved pain management and restoration of joint function compared to knee cavity injections based solely on surface anatomy positioning.^[37]^ Primarily, this is attributed to the enhanced injection accuracy facilitated by ultrasound guidance, ensuring tip placement away from pain-sensitive tissue. Moreover, factors such as the cooling effect of the coupler, pressure from the ultrasound probe, and distraction provided by patient observation of the ultrasound image also contribute to this outcome. Furthermore, in patients without joint effusion, injections based solely on body surface anatomical positioning may be inaccurate, resulting in increased pain and discomfort for the patient.^[51]^ The intra-articular injection of sodium hyaluronate and glucocorticoids into the knee joint has been demonstrated to yield favorable therapeutic outcomes. The effectiveness of sodium hyaluronate primarily lies in its ability to repair the physiological barrier, act as a molecular sieve, promote the synthesis of sodium hyaluronate, and stabilize pain receptors.^[52]^ Meanwhile, glucocorticoids exert a significant anti-inflammatory effect, effectively alleviating pain, with rapid and long-lasting effects.^[53]^ Ultrasound boasts high diagnostic accuracy, affordability, and ease of operation, rendering it advantageous for various medical procedure.^[54,55]^ MRI is a widely utilized clinical diagnostic method, renowned for its high sensitivity and specificity. However, it tends to be costly. In contrast, ultrasound is noninvasive, cost-effective, and convenient, offering the added benefit of good repeatability.^[56]^ Using ultrasound-guided arthrocentesis for knee disease has been extensively documented in the literature.^[57,58]^ In recent years, ultrasound has gained increasing prominence in the field of pain management, with its utilization becoming more widespread. Ultrasound serves as real-time guided imaging, enabling the observation of anatomy and lesions in various parts of the knee joint, providing valuable into injury conditions. In clinical practice, the injection site is typically determined by palpating the body surface mark. Nevertheless, when employing blind arthrocentesis, doctors can never ascertain the depth to which the needle is being inserted. Moreover, the accuracy of blind injection is notably deficient, particularly in obese patients lacking distinct anatomical landmarkers. Ultrasound-guided arthrocentesis has gained widespread adoption in recent years due to its radiation-free nature, supplanting conventional blind arthrocentesis, also known as the “blind” approach.^[58]^ The noninvasive characteristic of ultrasound allows for its repeated use without any adverse effects. Musculoskeletal ultrasound is characterized by its affordability, rapidity and repeatability. It enables dynamic monitoring of injuries and prognosis, making it valuable tool worthy of enthusiastic promotion. The real-time capability of ultrasound allows clinicians to observe the movement of tendons and muscles, a crucial aspect often challenging to discern with other imaging modalities. Ultrasound presents no evident contraindications, avoids radioactive harm, offers straightforward operation, and boasts low costs. The convenience of ultrasound enables its use in various settings, including emergency rooms, bedside examinations, operating room, and sports venues. This versatility further expands the application range of ultrasound. The high resolution of ultrasound allows for the simultaneous inspection of multiple parts and facilitates bilateral comparison, enhancing diagnostic capabilities. Ultrasound serves as a safe and precise technique, guiding punctures and penetration while ensuring the accurate placement of needles and delivery of medication. Abdel-Rahman Aly et al^[59]^ reported that ultrasound-guided injections demonstrated greater accuracy in all shoulder girdle injections except the subacromial space. They also suggested a greater degree of pain reduction in the ultrasound group and improvement in function in the subacromial space for the shoulder joint at 6 weeks. In addition, other studies^[22,60,61]^ have similarly concluded that ultrasound-guided injections can effectively reduce pain or enhance joint function compared to blind injections. Some systematic reviews^[7,24]^ have demonstrated that ultrasound-guided knee injections are more accurate at each anatomic needle injection site compared to blind injections. These findings are consistent with the conclusions drawn in our study. In addition, our study delved into aspects such as satisfaction, volume of fluid obtained, thickened synovial membrane, and costs. To date, comprehensive descriptions of these factors are lacking in existing literature, making our study unique in this regard.

### 4.1. Limitations to this systematic review

This review focuses on the latest advancements in knee arthrocentesis guided by ultrasound. It has been registered in PROSPERO. Studies were conducted in accordance with PRISMA statement guidelines strictly. Furthermore, we explore either our meta-analysis of the evidence is reliable and conclusion-independence by using TSA or not. Additionally, it is important to acknowledge that our study comes with certain limitations. Firstly, it is important to note that the duration of symptoms and sex ratio exhibit considerable variability. Secondly, it is noteworthy that some of the included trials had unclear risks of bias. Thirdly, the limited number of studies on satisfaction, volume of fluid obtained, thickened synovial membrane, and costs presents a limitation, as the existing evidence is insufficient. Fourthly, the scale we use to assess outcomes can be influenced by subjective assessments, such as pain, satisfaction, etc. Finally, our study is confined to English and Chinese languages, potentially excluding relevant literature in other languages.

## 5. Conclusions

When knee osteoarthritis is present, ultrasound-guided arthrocentesis may lead to greater clinical improvement compared to blind arthrocentesis. Due to the small sample sizes and limited number of participants in the study, it is imperative that we carefully interpret the findings. Low-quality evidence underscores the need for larger RCTs.

## Author contributions

**Conceptualization:** Xiaoyan Deng.

**Data curation:** Xiaoyan Deng.

**Investigation:** Daishun Li.

**Methodology:** Daishun Li.

**Software:** Xiaoyan Deng, Yamei Li.

**Visualization:** Daishun Li.

**Writing – original draft:** Daishun Li.

**Writing – review & editing:** Yamei Li, Daishun Li.

## Supplementary Material


