# Supplementary material for: Ultrasound-guided versus blind arthrocentesis in knee osteoarthritis: A systematic review and meta-analysis
Source: Medicine (Baltimore). 2025 Jan 31;104(5):e41389. doi: 10.1097/MD.0000000000041389 (PMC11789915; doi:10.1097/MD.0000000000041389)
Supplement: Supplementary file 1 [file medi-104-e41389-s001.docx]

**Supplementary Table 1: Search strategy**

**Final search strategy for PubMed**

| #ID | Topic or  intervention | Query | Records |
| --- | --- | --- | --- |
| #1 | Disease | (Osteoarthritis, Knee[MeSH Terms]) OR (Knee Osteoarthritis[MeSH Terms])) OR ((((((((Knee Osteoarthritis[Title/Abstract])) OR (Knee Osteoarthritides[Title/Abstract])) OR (Osteoarthritis of Knee[Title/Abstract])) OR (Osteoarthritis of the Knee[Title/Abstract])) OR (Knee OA[Title/Abstract])) OR (Knees OA[Title/Abstract])) OR (Osteoarthritis of Knees[Title/Abstract]))) OR ((((Knee[Title/Abstract])) OR (Knees[Title/Abstract])) AND (((((Osteoarthritis[Title/Abstract])) OR (Osteoarthritides[Title/Abstract])) OR (Arthritis[Title/Abstract])) OR (Arthritides[Title/Abstract])))  OR (gonarthrosis[Title/Abstract])) | 55458 |
| #2 | Study design | "Randomized Controlled Trial" [Publication Type] OR Randomized [Title/Abstract] OR Randomised [Title/Abstract] OR Randomization [Title/Abstract] OR Randomisation [Title/Abstract] | 1023400 |
| #3 | Intervention | (ultrasonography[MeSH Terms])) OR (ultrasound[Title/Abstract])) OR (Ultrasonic Imaging[Title/Abstract])) OR (Imaging, Ultrasonic[Title/Abstract])) OR (Ultrasonographic Imaging[Title/Abstract])) OR (Ultrasonic Tomography[Title/Abstract])) OR (ultrasonography[Title/Abstract])) AND (((Injections[MeSH Terms])) OR ((Injection[Title/Abstract]) | 546077 |
| #4 | Final query | #1 AND #2 AND #3 | 43611 |

**Final search strategy for Embase**

| #ID | Topic or  intervention | Query | Records |
| --- | --- | --- | --- |
| #1 | Disease | 'osteoarthritis, knee'/exp OR ' Knee OA ': ti, ab, kw OR ' knee osteoarthritis': ti, ab, kw ' 'osteoarthritis of knee': ti,ab,kw 'OR knee oa':ti,ab,kw' OR gonarthrosis ':ti,ab,kw | 42350 |
| #2 | Study design | 'randomized controlled trial'/exp OR 'randomized controlled trial': ti, ab, it OR 'randomized': ti,ab, it OR 'randomization': ti, ab, it OR rct : ti, ab, it | 1272348 |
| #3 | Intervention | ('injection'/exp OR injection: ab, ti, kw OR injections:ti,ab,kw) AND('ultrasound'/exp OR ultrasound: ab, ti, kw OR 'echography'/exp OR echography: ab, ti, kw) | 569860 |
| #4 | Final query | #1 AND #2 AND #3 | 3678 |

**Final search strategy for Web of Science**

| #ID | Topic or  intervention | Query | Records |
| --- | --- | --- | --- |
| #1 | Disease | TS= (knee osteoarthritis) OR TS= (gonarthrosis) | 59830 |
| #2 | Study design | TS= (Randomized controlled trial) | 363414 |
| #3 | Intervention | (TS= ultrasound) AND (TS=injection) | 9862 |
| #4 | Final query | #1 AND #2 AND #3 | 89 |

**Final search strategy for Cochrane Central Register of Controlled Trials**

| #ID | Topic or  intervention | Query | Records |
| --- | --- | --- | --- |
| #1 | Disease | MeSH descriptor: [[Osteoarthritis, Knee] explode all trees | 4903 |
| #2 |  | (Osteoarthritis, Knee): ti, ab, kw | 13942 |
| #3 |  | (knee osteoarthritis): ti, ab, kw | 13942 |
| #4 |  | (Knee OA: ti, ab, kw | 5052 |
| #5 |  | (osteoarthritis of knee): ti, ab, kw | 13734 |
| #6 |  | #1 OR #2 OR #3 OR #4OR #5 | 14043 |
| #7 | Study design | MeSH descriptor: [Randomized Controlled Trial] explode all trees | 119 |
| #8 |  | MeSH descriptor: [Random Allocation] explode all trees | 20669 |
| #9 |  | (randomized controlled trial): ti, ab,kw | 722330 |
| #10 |  | (random allocation): ti, ab, kw | 92908 |
| #11 |  | #7 or #8 or #9 or #10 | 759984 |
| #12 |  | #6 AND #11 | 7685 |
| #20  #21 | Intervention | (ultrasound): ti, ab, kw AND  MeSH descriptor: [Injections] explode all trees OR (Injections): ti, ab, kw | 33905  109558 |
| #22 |  | #20 AND #21 | 5401 |
| #23 | Final query | #12 AND #19 AND #22 | 23 |

**Final search strategy for** **Scopus**

| #ID | Topic or  intervention | Query | Records |
| --- | --- | --- | --- |
| #1 | Disease | TITLE-ABS-KEY ("Knee Osteoarthritis" )  OR TITLE-ABS-KEY ("Knee Osteoarthritides" )   OR TITLE-ABS-KEY ("Osteoarthritis of Knee" )   OR TITLE-ABS-KEY ("Knee OA" ) | 7443 |
| #2 | Study design | (TITLE-ABS-KEY ("Randomized Controlled Trial ") OR TITLE-ABS-KEY ("Randomized”) OR TITLE-ABS-KEY ("Randomization") OR TITLE-ABS-KEY ("Randomization") | 1282491 |
| #3 | hyaluronic acid | (TITLE-ABS-KEY ("hyaluronic acid  ") OR TITLE-ABS-KEY ("amo vitrax  ”) OR TITLE-ABS-KEY ( "hyaluronan" )  OR  TITLE-ABS-KEY ( " Hyaluronate Sodium" ) | 325740 |
| #4 | Intervention | (TITLE-ABS-KEY “ultrasound”) AND (TITLE-ABS-KEY” injection”) | 18392 |
| #5 | Final query | #1 AND #2 AND #3 AND #4 | 7 |

**Final search strategy for CNKI**

| #ID | Topic or  intervention | Query | Records |
| --- | --- | --- | --- |
| #1 | Disease | 主题：膝关节病 | 487 |
| #2 | Study design | 主题：随机对照试验 | 15600 |
| #3 | Intervention | 主题：超声下引导注射 | 1443 |
| #4 | Final query | #1 AND #2 AND #3 | 89 |

**Supplementary Table 2: Hierarchy of tools for patient-reported outcomes assessment**

| **Pain outcome** |
| --- |
| 1.Pain with activity (VAS) |
| 2.Pain at night (VAS) |
| 3.Global pain (VAS) |
| 4.Pain at rest (VAS) |
| 5.Pain subscale of SPADI |
| 6.Pain unspecified |
| **Function outcome (activity)** |
| 1. WOMAC |
| 2.the 36-item Short-Form Health Survey (SF-36) |
| 3. HAQ (disability subscale) |
| 4. ASES (disability subscale) |
