# Supplementary material for: Ultrasound-guided versus blind arthrocentesis in knee osteoarthritis: A systematic review and meta-analysis
Source: Medicine (Baltimore). 2025 Jan 31;104(5):e41389. doi: 10.1097/MD.0000000000041389 (PMC11789915; doi:10.1097/MD.0000000000041389)

Supplementary Figure 1

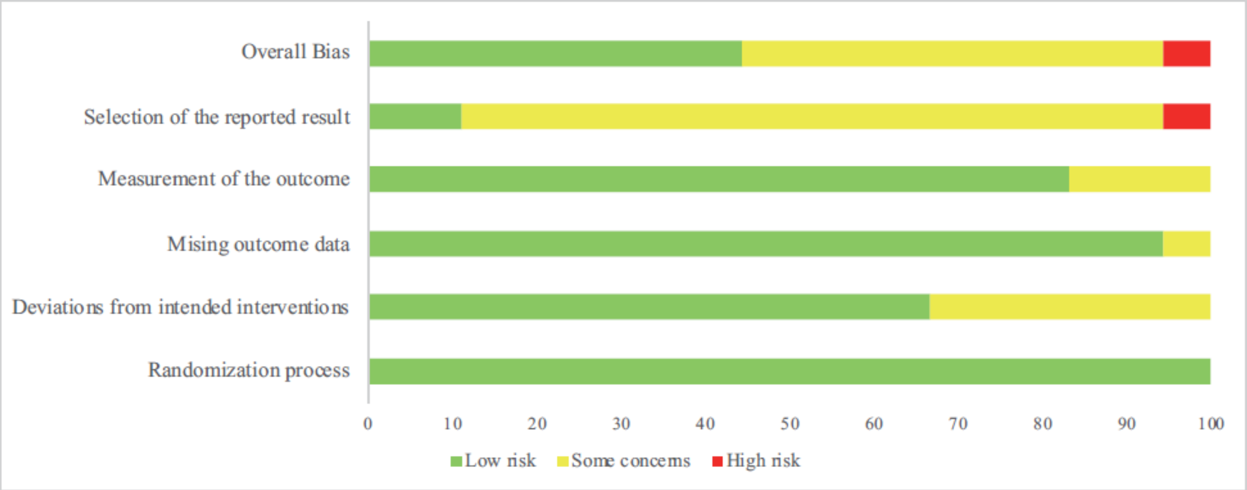

Supplementary Figure 2

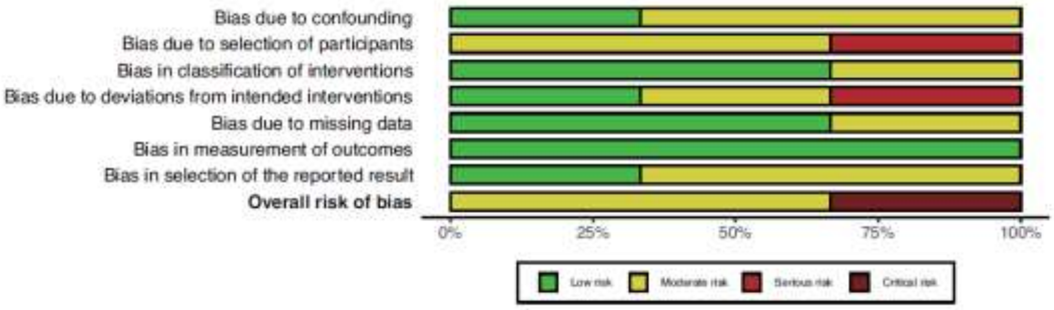

Supplementary Figure 3

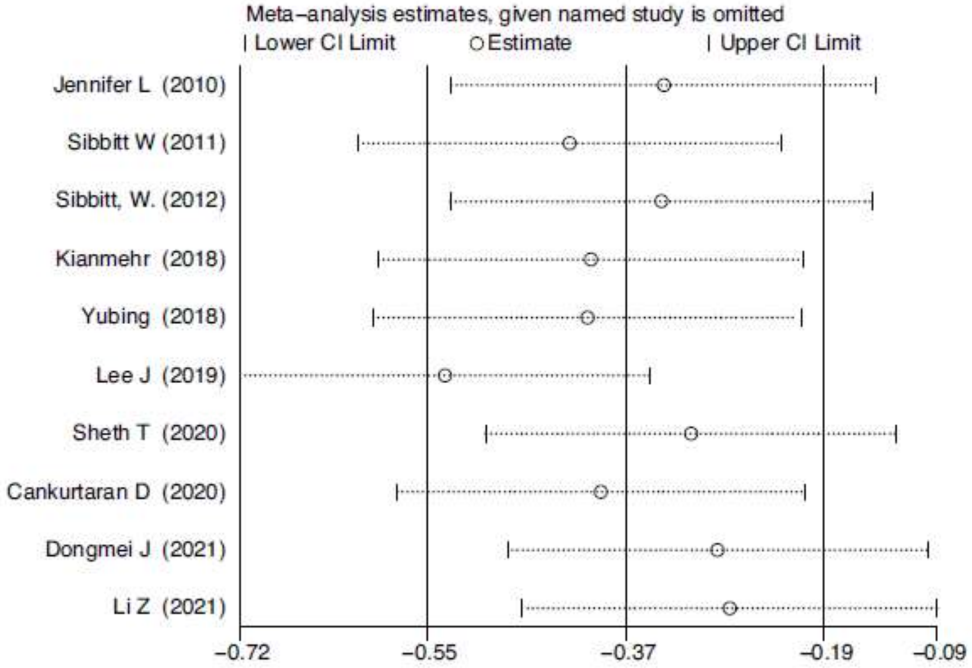

Supplemental Figure 4

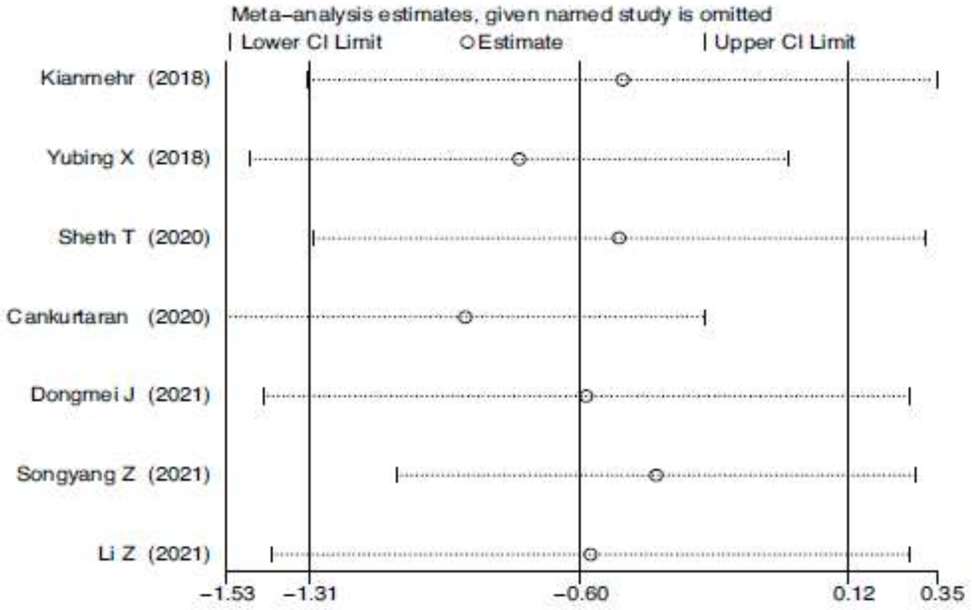

Supplementary Figure 5

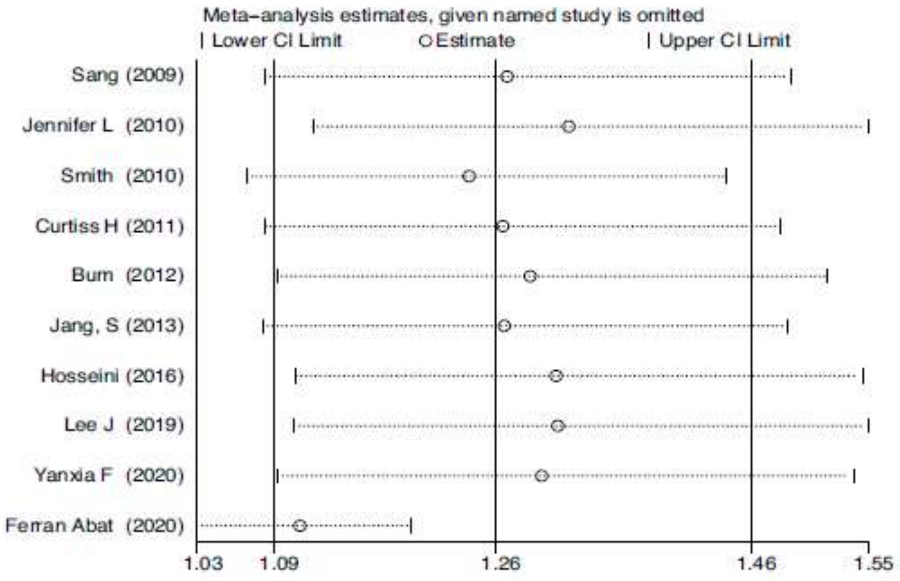

Supplementary Figure 6

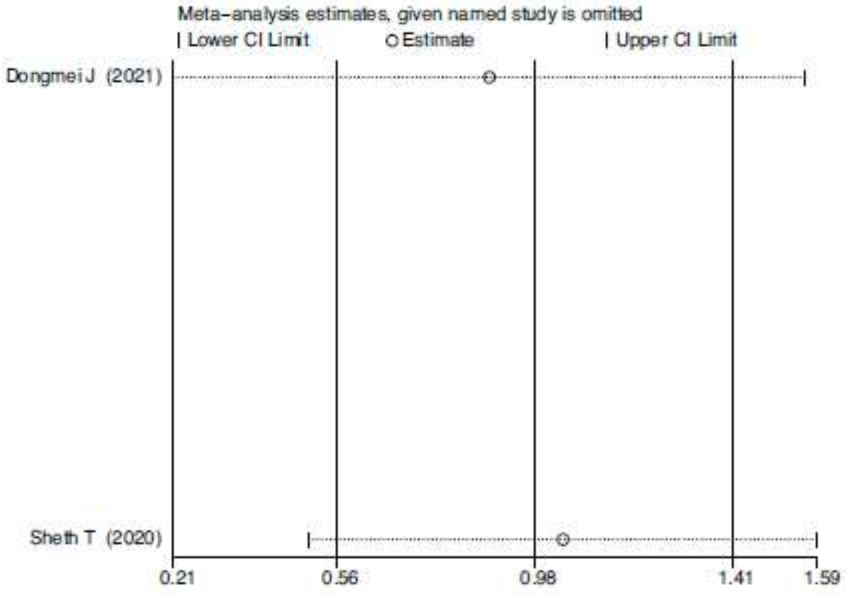

Supplementary Figure 7

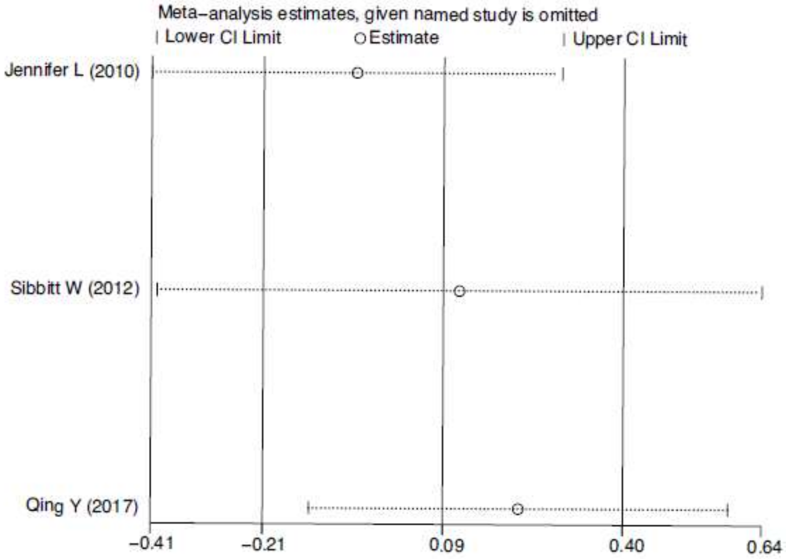

Supplementary Figure 8

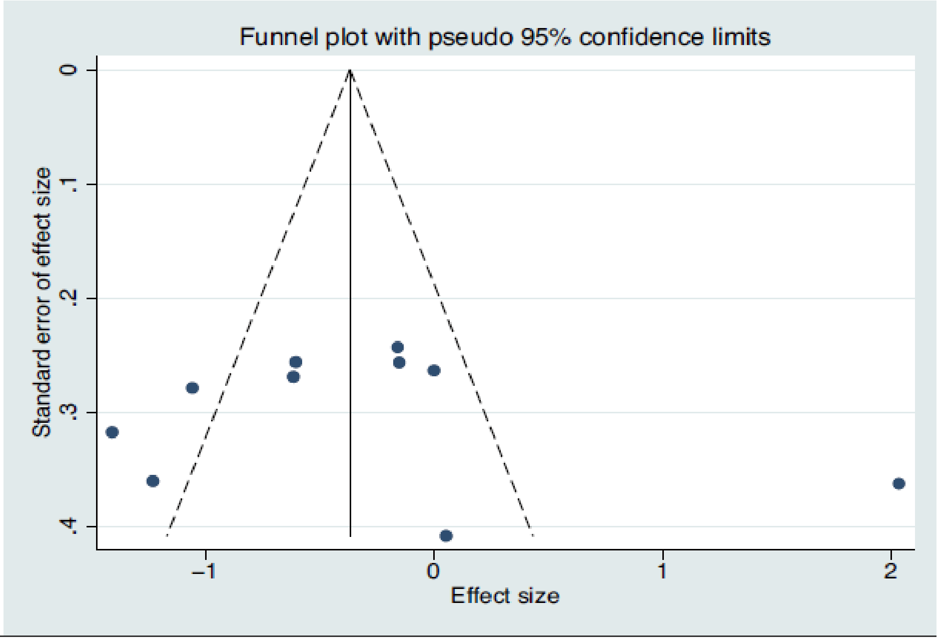

Supplementary Figure 9

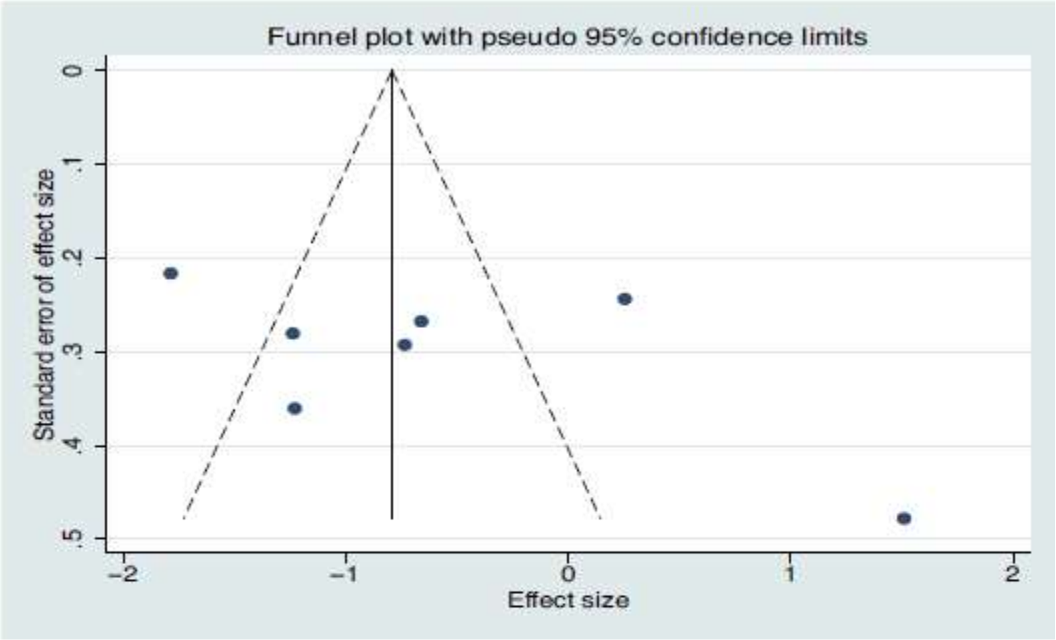

Supplementary Figure 10

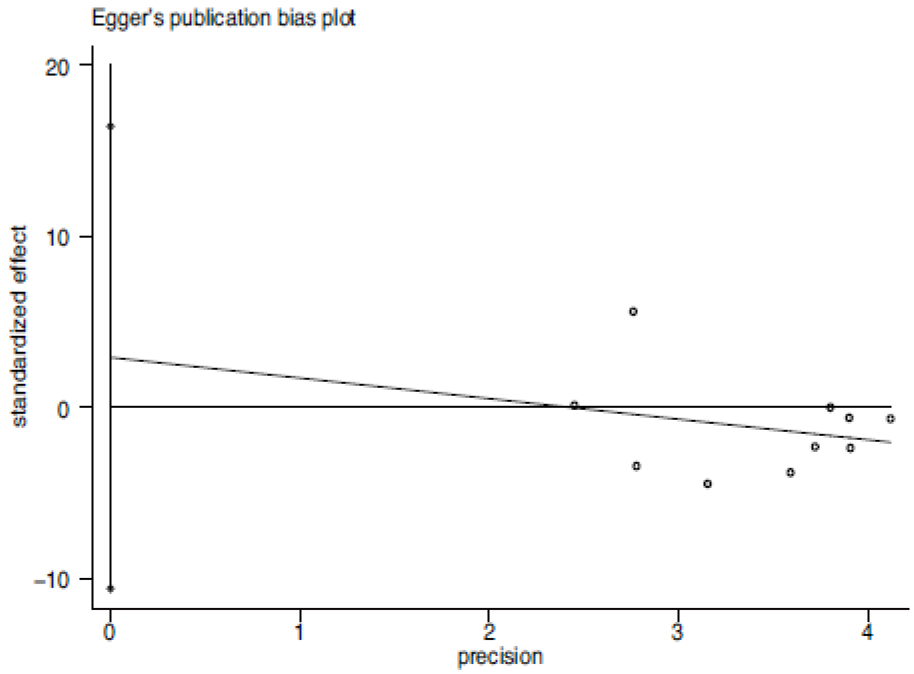

Supplementary Figure 11

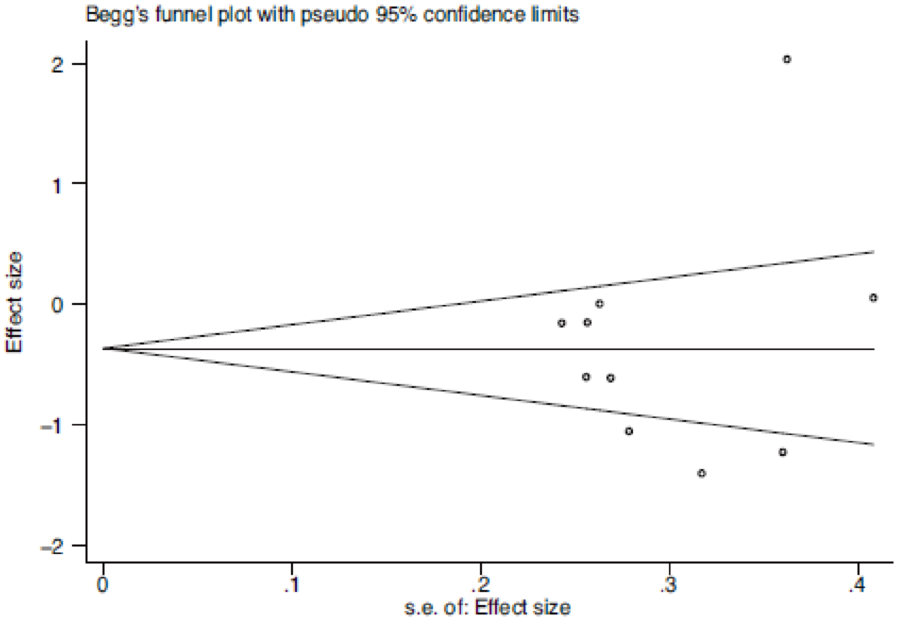

Supplementary Figure 12

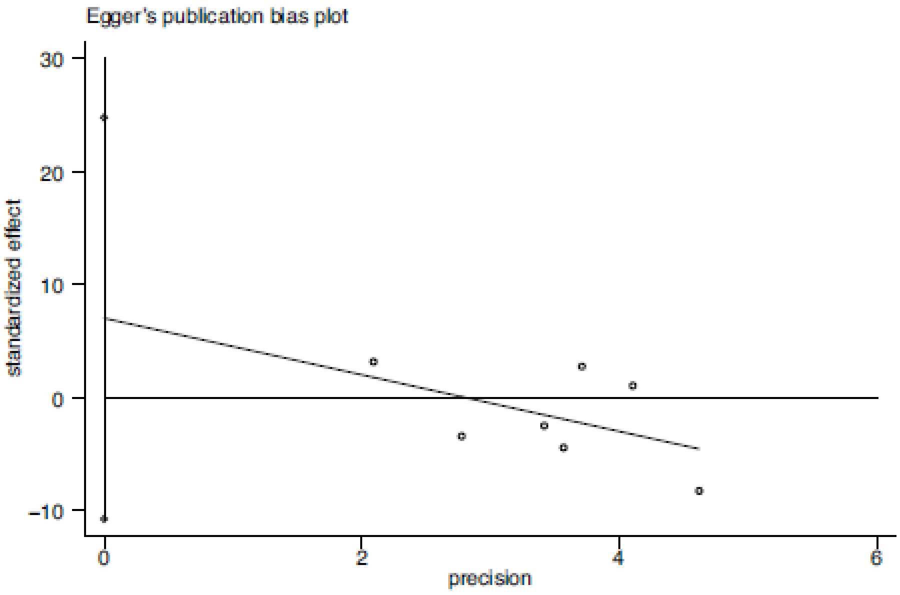

Supplementary Figure 13

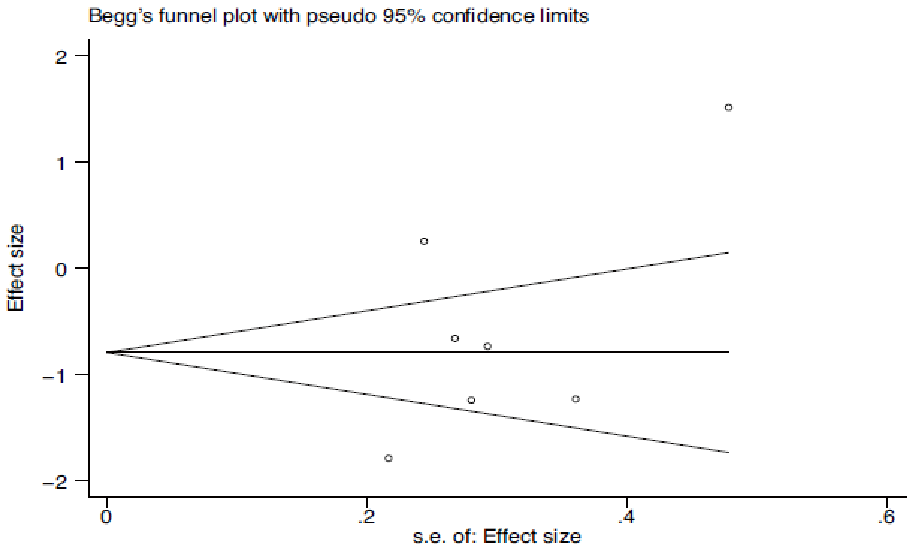

Supplementary Figure 14

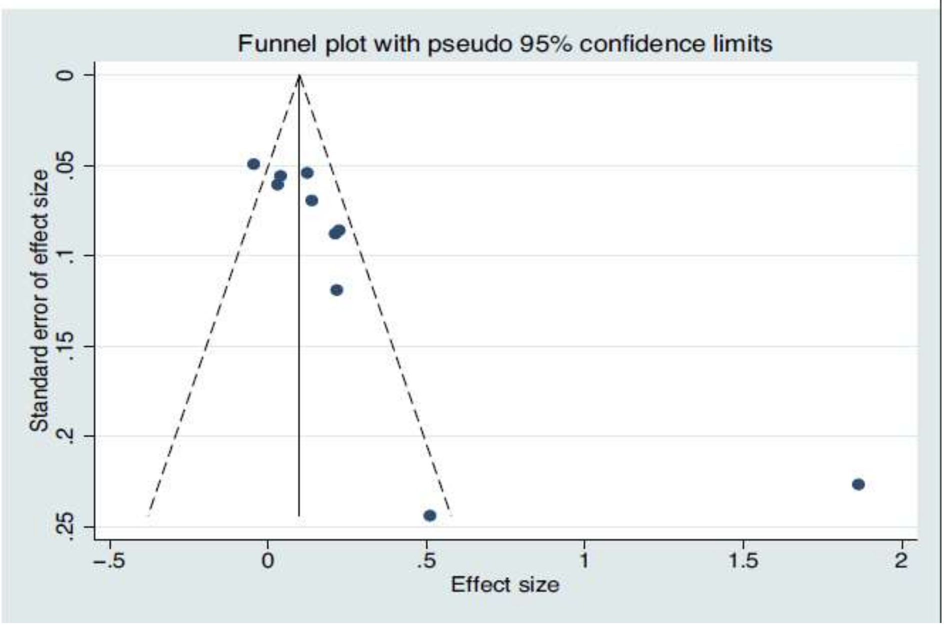

Supplementary Figure 15

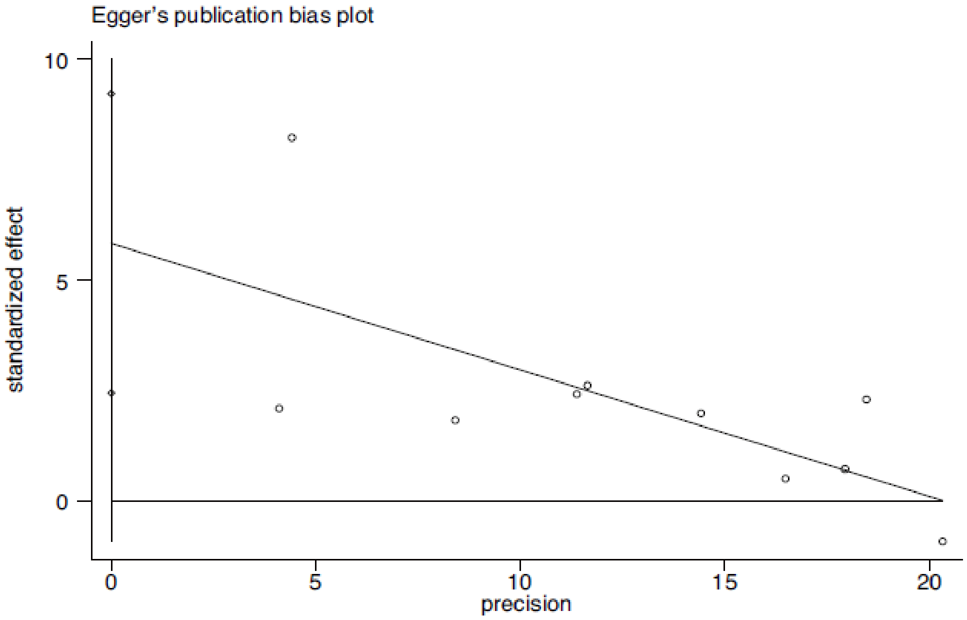

Supplementary Figure 16

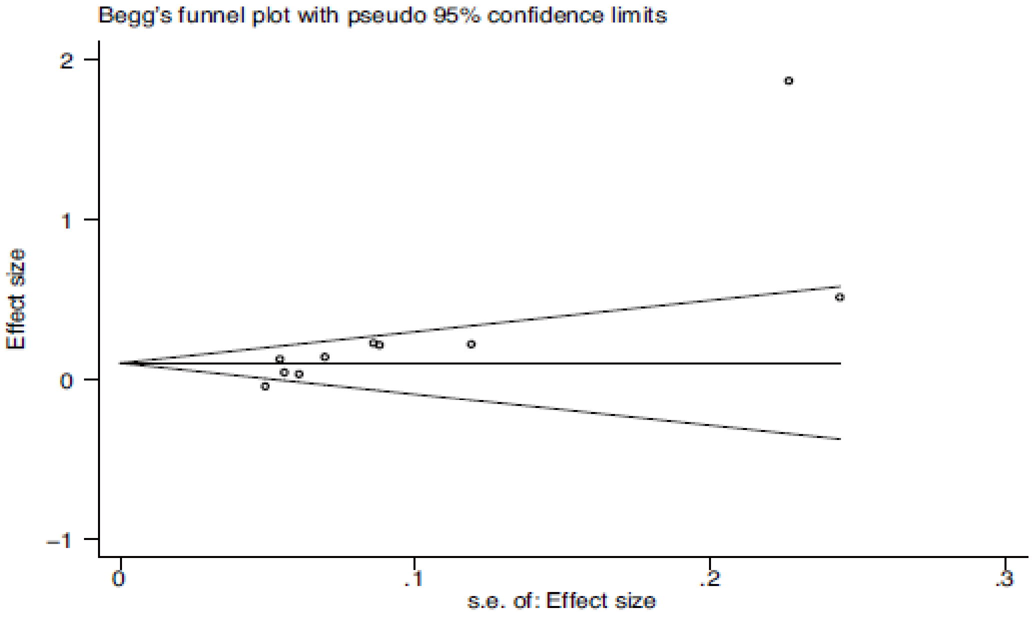

Supplementary Figure 17

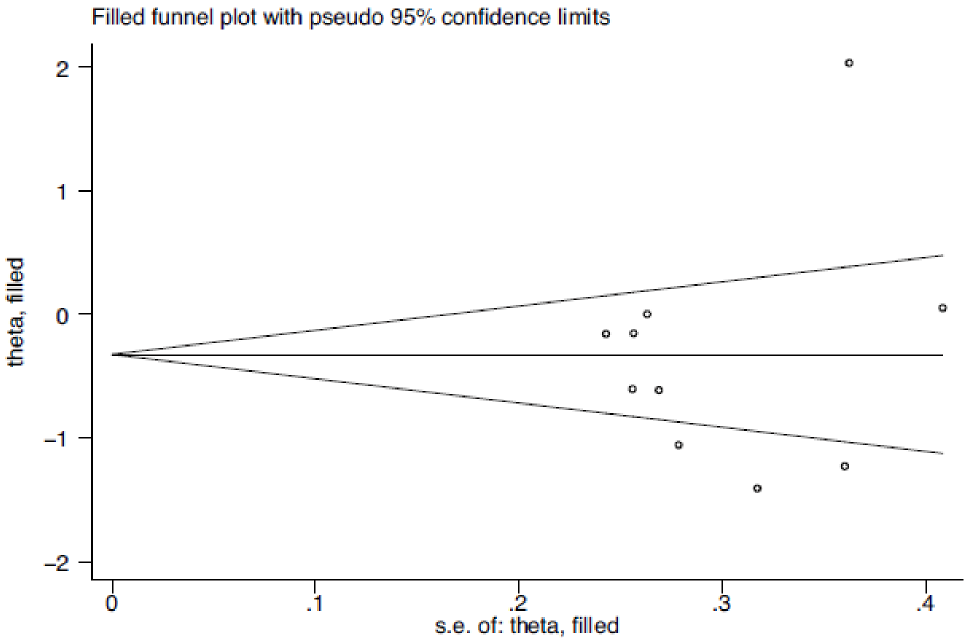

Supplementary Figure 18

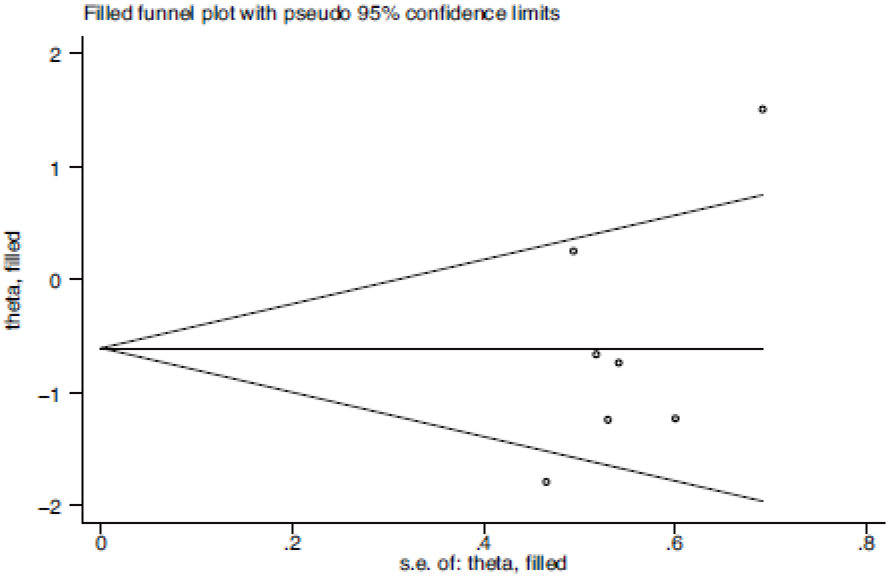

Supplementary Figure 19

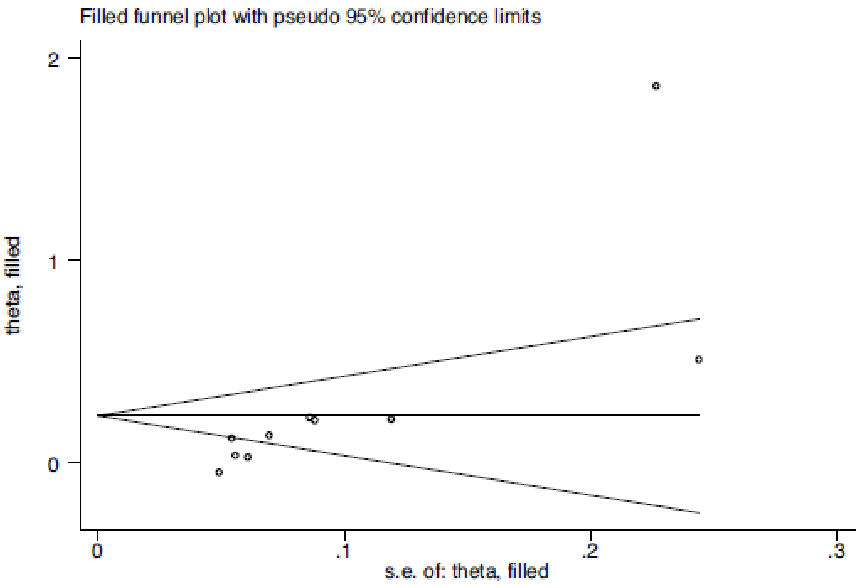

Supplementary Figure 20

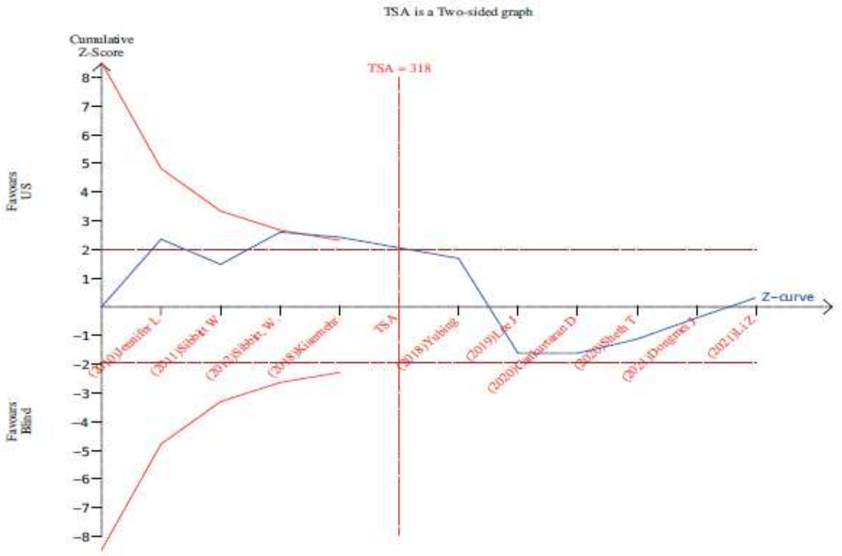

Supplementary Figure 21

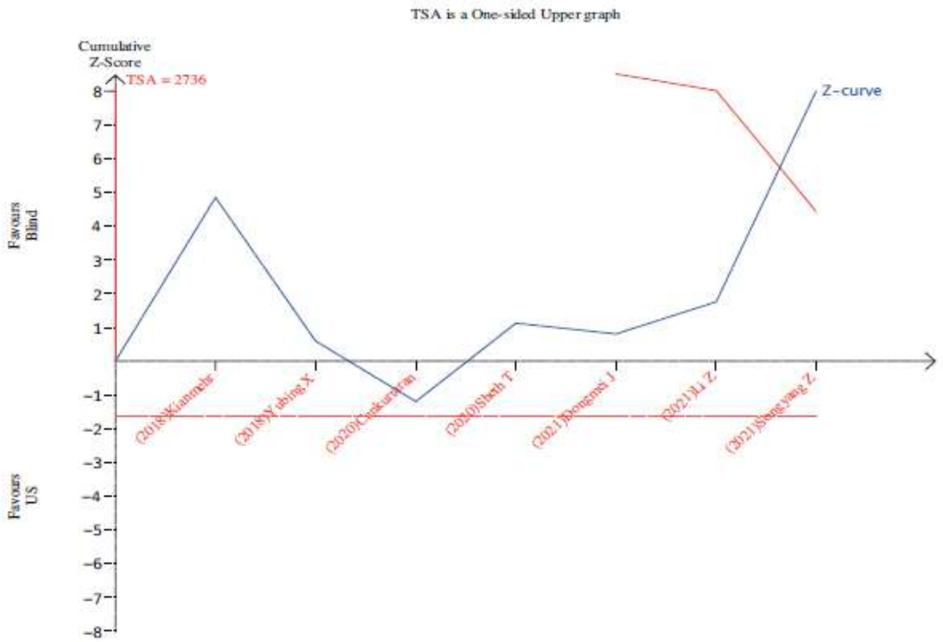

Supplementary Figure 22

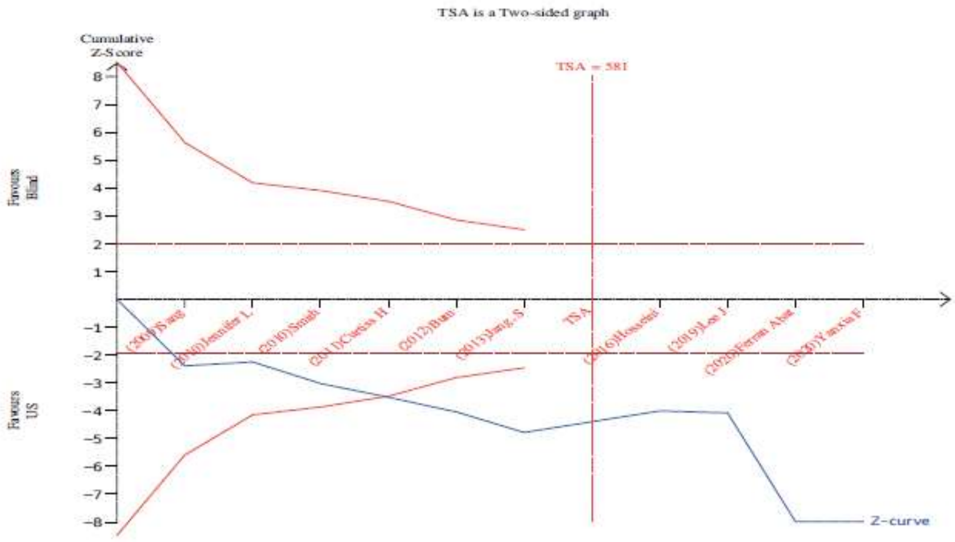

Supplement: Supplementary file 2 [file medi-104-e41389-s002.pdf]
